# Single intake of matcha increases brown adipose tissue activity in young women with low thermogenesis

**DOI:** 10.14814/phy2.70896

**Published:** 2026-05-01

**Authors:** Hirokazu Taniguchi, Chisaki Hachikawa, Sana Iwase, Shinsuke Nirengi, Tomomi Nagahata

**Affiliations:** ^1^ Faculty of Agriculture Ryukoku University Otsu Shiga Japan; ^2^ Division of Applied Life Sciences, Graduate School of Life and Environmental Sciences Kyoto Prefectural University Kyoto Japan; ^3^ Clinical Research Institute, Division of Preventive Medicine National Hospital Organization Kyoto Medical Center Kyoto Japan; ^4^ Dorothy M. Davis Heart and Lung Research Institute The Ohio State University Wexner Medical Center Columbus Ohio USA

**Keywords:** brown fat, crossover trial, Matcha, thermogenesis, young women

## Abstract

Matcha contains several bioactive compounds that contribute to the facilitation of thermogenesis. However, the effects of a single intake of matcha on brown adipose tissue (BAT) activity remain to be elucidated. This study aimed to evaluate the association between matcha intake and BAT activity. Healthy young women (*n* = 30) were enrolled in a randomized single‐blind crossover trial. Participants consumed 3 g of matcha powder or a placebo and then received the alternate condition in a subsequent trial. BAT activity was analyzed using thermography with cold exposure. A stratified analysis was performed based on tertiles of maximal changes in BAT activity. BAT activity was increased for all participants following cold exposure; however, no significant difference in the increased levels was found between the matcha and placebo trials. Stratified analysis showed that BAT activity was significantly different in the placebo trial across the low, middle, and high tertile groups; however, this was not found in the matcha trial. Differences in maximal BAT activity between the matcha and placebo trials were significantly higher in the low activity group than in the other groups. These results suggest that matcha powder has beneficial effects on BAT thermogenesis in young women with lower BAT activity.

## 1 INTRODUCTION

Brown adipose tissue (BAT) converts metabolic energy from glucose and fat into heat, thereby increasing energy expenditure (Kajimura & Saito, 2014; Sidossis & Kajimura, 2015). Previous studies reported that BAT depots are lower in obese and older individuals (Cypess et al., 2009; Nirengi & Stanford, 2023; Vijgen et al., 2011). In adult populations, higher BAT levels are associated with preferable cardiometabolic parameters (Becher et al., 2021; Chondronikola et al., 2014; Matsushita et al., 2014). The heat production of BAT is activated by cold exposure and increased glucose disposal (Hanssen et al., 2015; Iwen et al., 2017). Sympathetic nervous system activation is an underlying mechanism of the cold‐induced activation of BAT (hereafter, BAT activity) (Kajimura & Saito, 2014; Sidossis & Kajimura, 2015). Dietary interventions that activate the sympathetic nervous system have been explored as approaches to augment BAT activity, with capsinoids reported to increase activation (Nirengi, Homma, et al., 2016; Yoneshiro et al., 2013).

Recent reviews reported the health benefits of matcha (Sokary et al., 2023; Ye et al., 2024). Matcha is a powdered Japanese green tea that is derived from the tea leaf (Horie et al., 2017). Matcha, which is consumed whole, contains beneficial bioactive compounds, including catechins, caffeine, and theanine (Sokary et al., 2023; Ye et al., 2024). The intake of matcha has been demonstrated to have a favorable impact on obesity and glycolipid profiles in mice fed a high‐fat diet (Luo et al., 2024; Zhou et al., 2020). Theanine has been reported to stimulate thermogenic gene expression in the BAT of rodents (He et al., 2021; Peng et al., 2021). Previous studies reported that both single and daily ingestion of beverages containing catechin increased energy expenditure in young males (Nirengi, Amagasa, et al., 2016; Yoneshiro et al., 2017).

These findings suggest that matcha facilitates BAT activation; however, the effects of a single intake of matcha on BAT activity remain to be elucidated. The aim of this study was to determine whether the single intake of matcha increases BAT activity in young females. In order to examine the effect of daily consumption on BAT, the dosage of matcha used was equivalent to a cup of matcha (3 g).

## 2 MATERIALS AND METHODS

### 2.1 Ethics approval and trial registration

The present study was approved by the Ethical Committee of Kyoto Prefectural University (approval number 330). All participants provided written informed consent before enrollment. The study is registered with the University Hospital Medical Information Network in Japan (number 000056251) and was conducted in accordance with the Declaration of Helsinki.

### 2.2 Study design and participants

**FIGURE 1:**
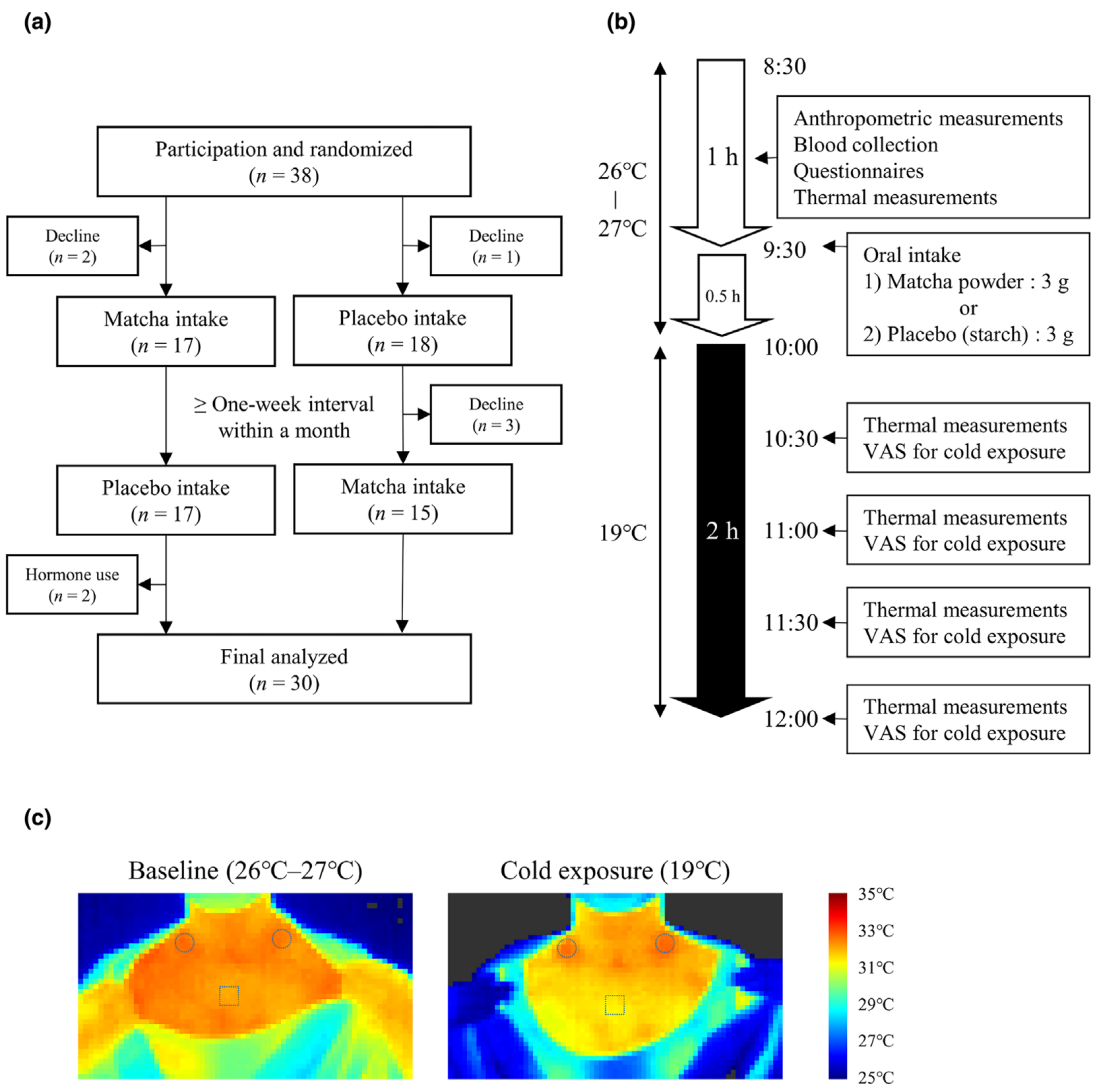
Study design. (a) Flow diagram of the participants. (b) Experimental flow of thermal measurements. VAS, visual analog scale. (c) Representative thermal images at baseline and during cold exposure. Circles indicate supraclavicular BAT depots, and squares indicate the manubrium.

We performed a randomized single‐blind crossover trial (Figure 1a). Thirty‐eight young healthy women (aged 18–25 years) participated in this study. The participants were recruited at Kyoto Prefectural University through flier and oral announcements. All participants had no history of chronic diseases or pregnancy. The participants were randomly assigned to either a trial that consumed matcha powder (*n* = 19) or placebo (*n* = 19). After randomization, three participants declined to participate; therefore, the first stage of the trial was performed for the remaining 35 participants. After the first placebo trial, three participants refused further participation. The remaining participants were switched to the other trial and performed the second trial after an interval of more than 7 days within a month. We further excluded two participants who used a hormonal drug for dysmenorrhea and analyzed the data of 30 participants.

### 2.3 Matcha powder and placebo

Matcha powder was obtained from Kyoeiseicha Co., Ltd. (Osaka, Japan). Each participant received 3 g of matcha powder that were individually wrapped in one‐gram portions using commercially available edible potato paper. The control consisted of potato starch colored with 3% commercially available green food coloring (Kyoritsu foods, Tokyo, Japan), which contains 8.4% food yellow no. 4, 3.6% food blue no. 1, and 88.0% dextrin, wrapped in one‐gram portions using commercially available edible potato paper. The composition of matcha powder was analyzed by the Japan Food Research Laboratories (Tokyo, Japan) and the Food Analysis Technology Center SUNATEC (Mie, Japan). Table 1 shows the composition of matcha and placebo (starch) powders. The nutrient content of the placebo was calculated according to the Standard Table of Food Composition in Japan (Ministry of Education, Culture, Sports, Science and Technology, 2023).

**TABLE 1:** Composition of matcha and placebo (starch) powders.

	Matcha (3 g)	Placebo (3 g)
Energy, kcal	9.5	10.1
Protein, g	0.8	0.0
Fat, g	0.2	0.0
Carbohydrate, g	0.6	2.4
Dietary fiber, g	0.9	0.0
Anhydrous caffeine, mg	90.0	
Vitamin K_1_, μg	93.0	
Vitamin C, mg	5.1	
Total Carotenoids, mg	4.5	
Lutein, mg	2.2	
Theanine, mg	45.0	
Polyphenol, g	0.4	
Epicatechin, mg	16.5	
Epicatechin Gallate, mg	24.0	
Epigallocatechin, mg	75.0	
Epigallocatechin gallate, mg	162.0	
Catechin, mg	1.3	
Gallocatechin, mg	3.3	
Gallocatechin gallate, mg	0.9	
Chlorophyll a, mg	19.5	
Chlorophyll b, mg	8.7	

### 2.4 Measurement of BAT activity and oral intake

BAT activity was assessed by thermal imaging in the morning (between 8:30 and 12:00) in December of 2024 and January of 2025 (Figure 1b) (Kataoka et al., 2024; Nirengi et al., 2019; Taniguchi et al., 2020). Although 18‐fluorodeoxyglucose positron emission tomography/computed tomography (18 FDG‐PET/CT) is the gold standard for assessing BAT activity in vivo (Kajimura & Saito, 2014; Sidossis & Kajimura, 2015), its use involves radiation exposure; therefore, infrared thermography was employed as a noninvasive alternative in this study. Infrared thermography is a promising method for detecting the activation of BAT (Brasil et al., 2020; Jang et al., 2014; Nirengi et al., 2019). A previous study reported that BAT was detected in 5%–10% of individuals under thermoneutral conditions, with detection rates increasing following cold exposure (Townsend et al., 2023). As infrared thermography has been used to detect the cold‐induced activation of BAT (Jang et al., 2014; Nirengi et al., 2019), this study examined the effects of matcha consumption on BAT activity under conditions of cold exposure.

All the participants were instructed to consume their evening meal prior to 21:00 on the previous day and to skip breakfast. Prior to the measurement, the participants changed into disposable screening wear (AD‐5; Navis, Osaka, Japan), which was worn over a thin tank top and shorts. Body surface temperature was measured using a thermal imaging camera (DETC1000T; D‐eyes, Osaka, Japan). Participants were seated at rest under thermoneutral conditions (26–27°C) for at least 30 min before baseline body surface temperature was measured. The participants consumed 3‐g samples of matcha or placebo, which were still wrapped, at 9:30. The samples were administered with water.

An artificial climate chamber (TBRR‐9A4GX; ESPEC, Osaka, Japan) set at 19°C was used for cold exposure. Participants entered the artificial climate chamber barefoot. Body surface temperature was measured at 30, 60, 90, and 120 min after mild cold exposure. Image acquisition was duplicated for each measurement. Before and during cold exposure, participants were asked to rate their cold sensation and perceived shivering using a visual analog scale (Kataoka et al., 2024; Nirengi et al., 2019; Taniguchi et al., 2020). In adult humans, BAT is mainly located in the supraclavicular region (Kajimura & Saito, 2014; Sidossis & Kajimura, 2015). Supraclavicular temperature was measured from a location adjacent to BAT on both the right and left sides from each image. Manubrium temperature was simultaneously measured as a control (Fuller‐Jackson et al., 2020; Taniguchi et al., 2024). Images of body surface temperature were analyzed using a modified (D‐eyes) version of the Thermal‐Cam v.1.1.0.9 software (Laon People, Seoul, Korea). Representative thermal images were demonstrated in Figure 1c. For duplicate images, the average temperature of nine pixels was calculated in both the supraclavicular and manubrial areas. The average supraclavicular temperature minus manubrium temperature was used to estimate BAT activity. To calculate changes in BAT activity (⊿ BAT activity), the baseline value was subtracted from the value measured after each cold exposure. Maximal BAT activity and maximal ⊿ BAT activity were defined as the highest BAT activity during cold exposure.

### 2.5 Anthropometric characteristics, dietary food intake, and questionnaire

Body weight and body fat percentage were measured using an electronic scale (Inbody Dial H20N; InBody Japan, Tokyo, Japan). Body mass index (BMI) was calculated as body weight (kg) divided by the square of height (m). Dietary intake was evaluated using the Brief‐type Self‐administered Diet History Questionnaire. The questionnaire is a paper‐based questionnaire designed for Japanese adults consisting of approximately 80 questions to assess dietary habits over the previous month. A dedicated calculation program was used to estimate individual nutrient intake and food intake across 15 food groups. Its validity has been previously confirmed (Kobayashi et al., 2011, 2012). Caffeine intake was estimated using the Standard Table of Food Composition in Japan (Ministry of Education, Culture, Sports, Science and Technology, 2023). Days of regular menstrual cycle, days after menstruation, and hormonal contraceptive use were evaluated using a self‐administered questionnaire.

### 2.6 Blood analysis

Blood samples were collected under thermoneutral conditions in both the matcha and placebo trials. Serum triglycerides, total cholesterol, low‐density lipoprotein cholesterol, high‐density lipoprotein cholesterol, and hemoglobin a1c were determined by Kyoto Biken Laboratories (Kyoto, Japan). The collected blood samples were centrifuged at 3000 rpm for 5 min, and the plasma was stored at −80°C until the time of analysis. Commercially available enzyme‐linked immunosorbent assay kits were used to measure plasma levels of progesterone (RE52231; IBL International GMBH, Hamburg, Germany), 17β‐estradiol (RE52041; IBL International GMBH), and fibroblast growth factor 21 (FGF21) (DF2100; R&D Systems, Minneapolis, USA). Concentrations of plasma free fatty acids and glucose were measured using LabAssay™ NEFA (FFA) and Glucose kits (299–94,301 and 291–94,001; FUJIFILM Wako Pure Chemical, Osaka, Japan), respectively.

### 2.7 Statistics

All statistical analyses were performed using SPSS, version 29.0 (SPSS, Chicago, USA). Kolmogorov–Smirnov test was performed to assess the normality of data distribution, and several variables were log or square root transformed prior to analysis to obtain a normal distribution of values. The data between each trial were compared using paired Student's *t*‐test for continuous variables or Chi‐square test for categorical variables. Two‐way ANOVA analysis (time×trial) was used to determine the difference of BAT activity between each trial. To determine the association between the degree of BAT activity and matcha intake, the participants were divided into tertile groups according to their maximal ⊿ BAT activity in a placebo trial. The difference in BAT activity between each time point of the matcha trial and that of the placebo trial was calculated, and the maximal difference was identified. The absolute differences among tertile groups were analyzed using one‐way ANOVA. Two‐way ANOVA (group or time×trial) with Tukey's post hoc analysis was used to determine differences in blood parameters and BAT activity among tertile groups. All measurements and calculated values are presented as the means ± standard deviations for continuous variables or as the number of cases and percentage for categorical variables. Significance was set at *p* < 0.05.

## 3 RESULTS

### 3.1 Participant characteristics at baseline and between trials

Table 2 shows the participant characteristics at baseline (the initial trial) and at each subsequent trial. The BMI range of the female participants was 16.9–24.7 kg/m^2^. Thirteen percent of the participants (*n* = 4) were categorized as underweight (BMI < 18.5 kg/m^2^). One‐third of the total participants (*n* = 10) had BMI < 20.0 kg/m^2^. There was no significant difference in the percentage of participants in the follicular phase between the matcha and placebo trials. Furthermore, there were no significant differences in blood glucose or lipid variables, plasma progesterone, and plasma FGF21 levels between the trials. Plasma 17β‐estradiol levels were slightly higher in the placebo trial than in the matcha trial.

**TABLE 2:** Characteristics at baseline and each trial of Japanese young females (*n* = 30).

	Baseline
Age, years	20.7 ± 1.5
Height, cm	159.5 ± 4.7
Weight, kg	53.0 ± 6.0
BMI, kg/m^2^	20.8 ± 1.8
Body fat, %	29.1 ± 3.7
Energy, kcal	1533 ± 335
Protein energy ratio, %	15.4 ± 1.8
Fat energy ratio, %	30.0 ± 5.7
Carbohydrate energy ratio, %	54.6 ± 6.8
Caffeine, mg	73.7 ± 70.4

	Placebo	Matcha	*p*
Follicular phase, *n* (%)	5 (16.7)	3 (10.0)	0.448
Triglycerides, mg/dL	54.3 ± 17.8	55.9 ± 20.7	0.618
Free fatty acids, mEq/L	0.29 ± 0.14	0.30 ± 0.15	0.774
Total cholesterol, mg/dL	185.1 ± 28.5	187.3 ± 28.1	0.481
LDL cholesterol, mg/dL	99.6 ± 21.7	98.7 ± 21.1	0.683
HDL cholesterol, mg/dL	67.3 ± 13.2	69.4 ± 12.7	0.110
Fasting glucose, mg/dL	92.7 ± 5.7	92.1 ± 5.0	0.536
HbA1c, %	5.4 ± 0.2	5.4 ± 0.2	0.489
Progesterone, ng/mL	6.8 ± 4.3	7.5 ± 4.0	0.497
17β‐estradiol, pg/mL	561.0 ± 199.6	474.7 ± 168.3	0.048
FGF21, pg/mL	116.9 ± 76.4	133.6 ± 140.5	0.302

*Note*: Data are the mean ± standard deviations (continuous variables) or *n* (%) (categorical variables). Significant difference between trials using the paired *t*‐test (continuous variables) or Chi‐square test (categorical variables).

Abbreviations: BMI, body mass index; FGF21, fibroblast growth factor 21; HbA1c, glycated hemoglobin A1c; HDL, high‐density lipoprotein; LDL, low‐density lipoprotein.

### 3.2 Effects of single matcha intake on BAT activity

**FIGURE 2:**
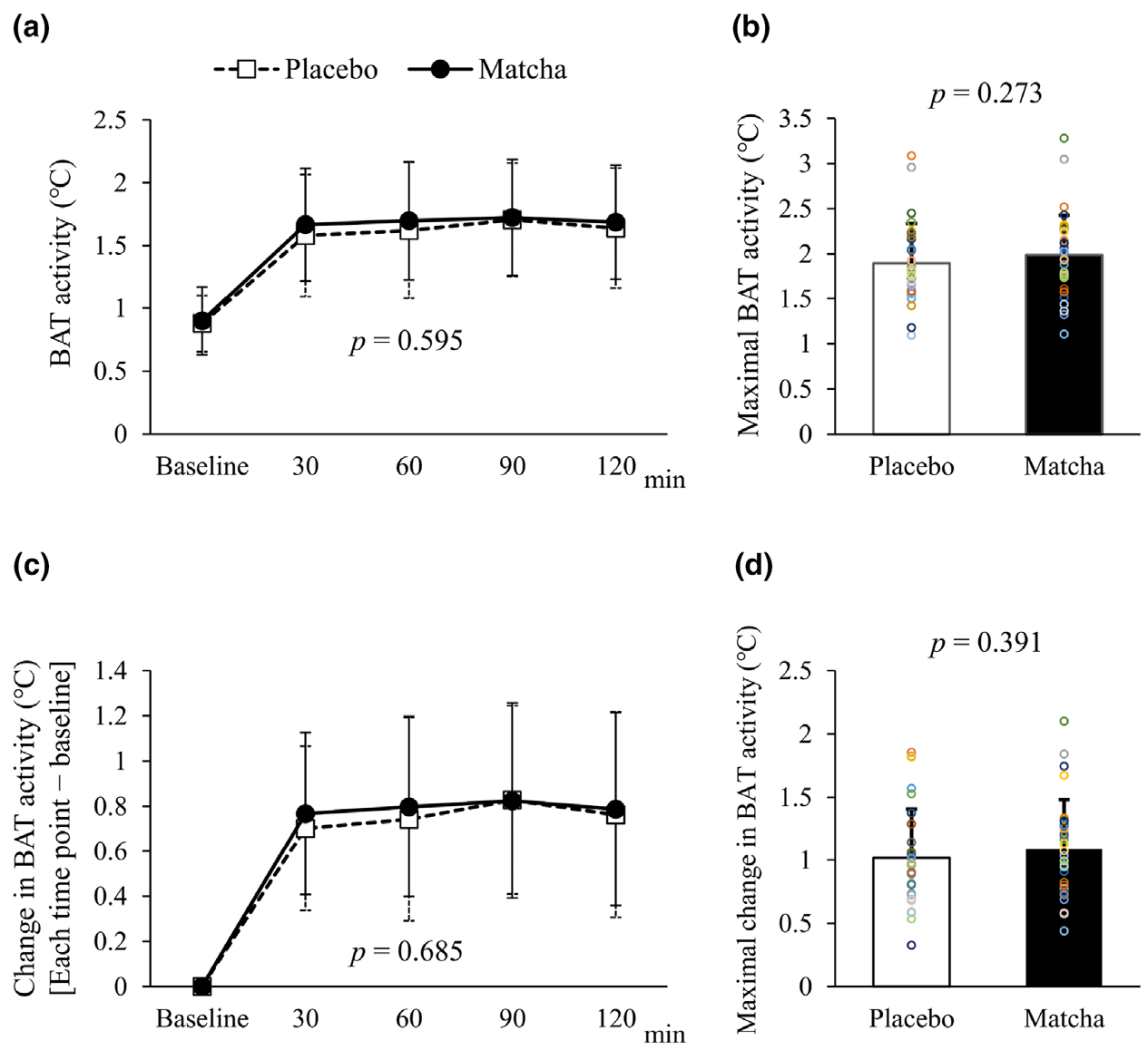
BAT activity in the placebo and matcha trials. (a) Time course of BAT activity before and during cold exposure. (b) Maximal BAT activity. The individual data points are indicated by color circles. (c) Time course of change in BAT activity before and during cold exposure. (d) Maximal change in BAT activity. The individual data points are indicated by color circles.

The time course of both raw and ⊿ BAT activity is shown in Figure 2a,c. At baseline, there was no difference in BAT activity between the trials. Although BAT activity was clearly elevated after cold exposure in both the placebo and matcha trials, there were no significant differences in the time course of BAT activity between the trials. There was no significant difference in raw and ⊿ maximal activity in BAT between the placebo and matcha trials (Figure 2b,d). Perceived shivering and cold sensation were not significantly different between the trials (data not shown).

### 3.3 Participant characteristics among tertile groups of ⊿ BAT activity

Table 3 shows the baseline characteristics of participants among tertile groups according to maximal ⊿ BAT activity in the placebo trial. BMI, body fat percentage, and dietary intake were not significantly different among the tertile groups. Menstrual cycle and blood parameters for each trial are shown in Table 4. In each trial, the proportion of participants in the follicular phase ranged from 10% to 20% across tertile groups. There were no significant differences in blood lipid or glucose parameters, and plasma levels of progesterone, 17β‐estradiol, and FGF21 among the tertile groups or between the trials.

**TABLE 3:** Characteristics at baseline of Japanese young females in tertile groups according to maximal changes in BAT activity.

	Low (tertile 1, *n* = 10)	Middle (tertile 2, *n* = 10)	High (tertile 3, *n* = 10)	*p*
Age, years	20.5 ± 1.2	20.7 ± 1.6	20.9 ± 1.9	0.853
Height, cm	158.0 ± 3.5	158.5 ± 6.3	162.0 ± 3.0	0.120
Weight, kg	52.5 ± 5.3	52.6 ± 7.4	53.9 ± 5.8	0.857
BMI, kg/m^2^	21.0 ± 1.6	20.9 ± 2.2	20.5 ± 1.9	0.836
Body fat, %	29.6 ± 3.2	28.4 ± 4.5	29.2 ± 3.4	0.765
Energy, kcal	1415 ± 401	1734 ± 282	1449 ± 231	0.060
Protein energy ratio, %	15.0 ± 2.4	15.8 ± 1.5	15.6 ± 1.7	0.616
Fat energy ratio, %	30.2 ± 7.8	29.8 ± 5.3	29.9 ± 4.1	0.990
Carbohydrate energy ratio, %	54.8 ± 9.4	54.4 ± 5.4	54.6 ± 5.4	0.989
Caffeine, mg	96.2 ± 96.3	60.3 ± 66.2	64.7 ± 38.1	0.476

*Note*: Data are the mean ± standard deviations. Significant difference among trials using one‐way ANOVA. The tertile groups were based on maximal changes in BAT activity in a placebo trial.

Abbreviation: BMI, body mass index.

**TABLE 4:** Characteristics at each trial of Japanese young females according to maximal changes in BAT activity.

	Low (tertile 1, *n* = 10)		Middle (tertile 2, *n* = 10)		High (tertile 3, *n* = 10)		*p*	
	Placebo	Matcha	Placebo	Matcha	Placebo	Matcha	Group	Intervention
Follicular phase, *n* (%)	2 (20.0)	1 (10.0)	1 (10.0)	1 (10.0)	2 (20.0)	1 (10.0)	0.787*	
Triglycerides, mg/dL	51.0 ± 14.0	59.0 ± 26.0	54.0 ± 26.0	50.0 ± 16.0	58.0 ± 11.0	59.0 ± 20.0	0.553	0.754
Free fatty acids, mEq/L	0.22 ± 0.07	0.30 ± 0.20	0.33 ± 0.12	0.33 ± 0.14	0.32 ± 0.19	0.26 ± 0.10	0.357	0.806
Total cholesterol, mg/dL	182.0 ± 32.0	189.0 ± 30.0	194.0 ± 34.0	194.0 ± 35.0	179.0 ± 17.0	180.0 ± 16.0	0.273	0.763
LDL cholesterol, mg/dL	96.0 ± 29.0	97.0 ± 24.0	104.0 ± 19.0	100.0 ± 24.0	99.0 ± 17.0	99.0 ± 17.0	0.721	0.866
HDL cholesterol, mg/dL	69.0 ± 14.0	72.0 ± 9.0	70.0 ± 15.0	73.0 ± 16.0	63.0 ± 11.0	64.0 ± 12.0	0.129	0.526
Fasting glucose, mg/dL	93.4 ± 5.8	92.7 ± 4.8	90.4 ± 4.9	91.3 ± 4.7	94.2 ± 6.0	92.2 ± 5.8	0.312	0.670
HbA1c, %	5.4 ± 0.3	5.5 ± 0.3	5.4 ± 0.2	5.4 ± 0.1	5.4 ± 0.2	5.4 ± 0.2	0.535	0.809
Progesterone, ng/mL	6.8 ± 4.3	8.5 ± 4.0	8.3 ± 4.3	6.1 ± 4.5	5.3 ± 4.1	8.1 ± 3.4	0.763	0.491
17β‐estradiol, pg/mL	545 ± 192	494 ± 166	602 ± 196	458 ± 157	536 ± 224	472 ± 195	0.911	0.084
FGF21, pg/mL	120.5 ± 51.2	110.1 ± 80.8	99.8 ± 97.4	118.2 ± 193.0	130.2 ± 78.5	172.4 ± 131.5	0.460	0.576

*Note*: Data are the mean ± standard deviations (continuous variables) or *n* (%) (categorical variables). Significant difference between trials using two‐way ANOVA (group × trial).

Abbreviations: FGF21, fibroblast growth factor 21; HbA1c, glycated hemoglobin A1c; HDL, high‐density lipoprotein; LDL, low‐density lipoprotein.

* Chi‐square test among tertile groups. The tertile groups were based on maximal changes in BAT activity in a placebo trial.

### 3.4 Comparison of BAT activity among the tertile groups

**FIGURE 3:**
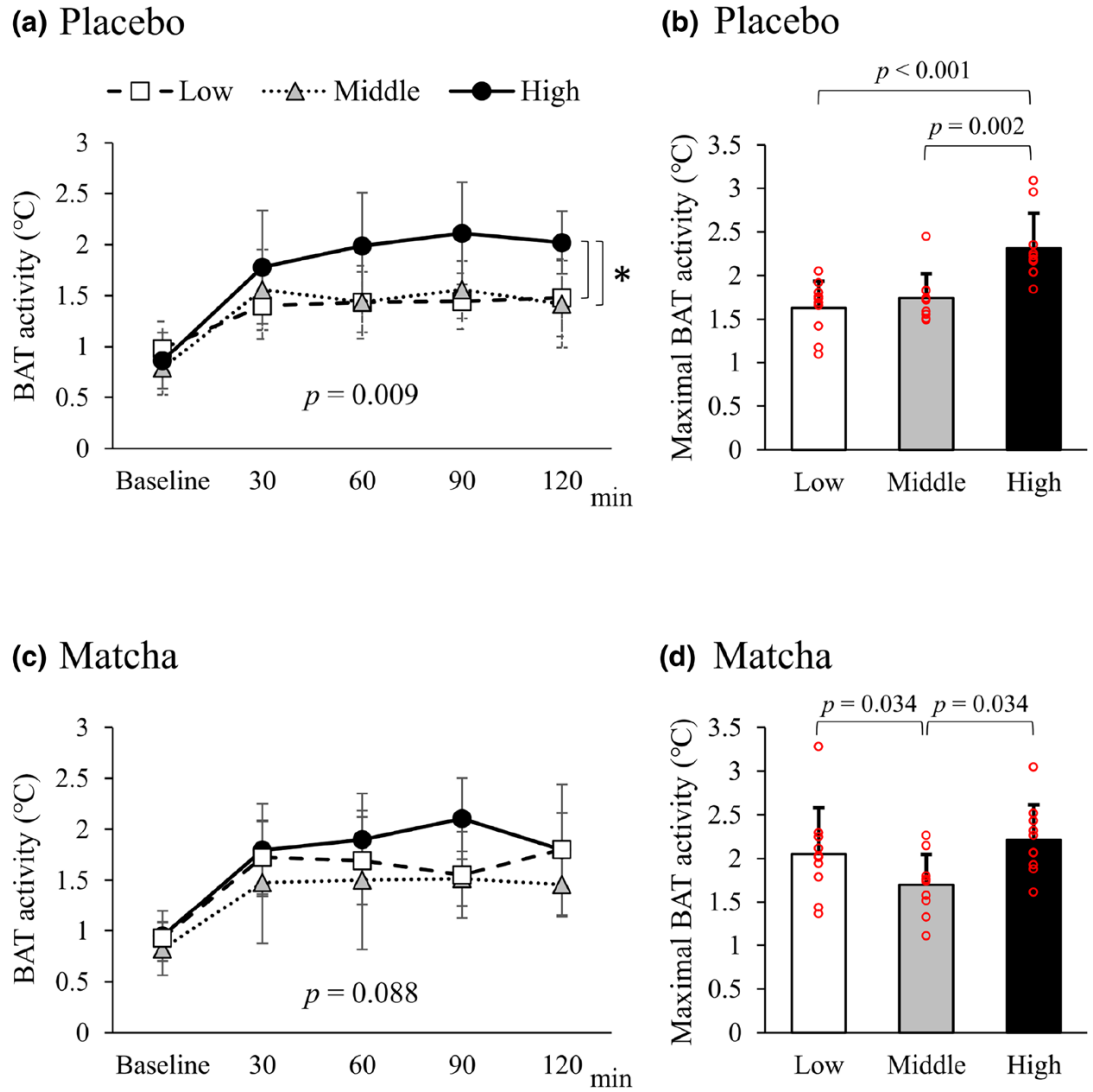
BAT activity in tertile groups according to maximal change in BAT activity. The tertile groups were based on maximal changes in BAT activity in a placebo trial. (a) Time course of BAT activity in the placebo trial. An asterisk indicate significant difference. (b) Maximal BAT activity in the placebo trial. The individual data points are indicated by red circles. (c) Time course of BAT activity in the matcha trial. (d) Maximal BAT activity in the matcha trial. The individual data points are indicated by red circles.

The time course of BAT activity and maximal BAT activity was compared among the tertile groups in each trial (Figure 3). Two‐way ANOVA analysis revealed that BAT activity was significantly higher in the high activity (third tertile) group than in the other tertile groups only after placebo intake (Figure 3a), but not after matcha intake (Figure 3c). In the placebo trial, significantly higher levels of maximal BAT activity were observed in the high activity group compared with the other tertile groups (Figure 3b). In the matcha trial, maximal BAT activity was significantly lower in the middle (second tertile) group than in the first and third tertile groups (Figure 3d). There was no significant difference in maximal BAT activity between the high and low tertile groups after matcha intake. Perceived shivering and cold sensation were not significantly different among the groups in each trial (data not shown).

**FIGURE 4:**
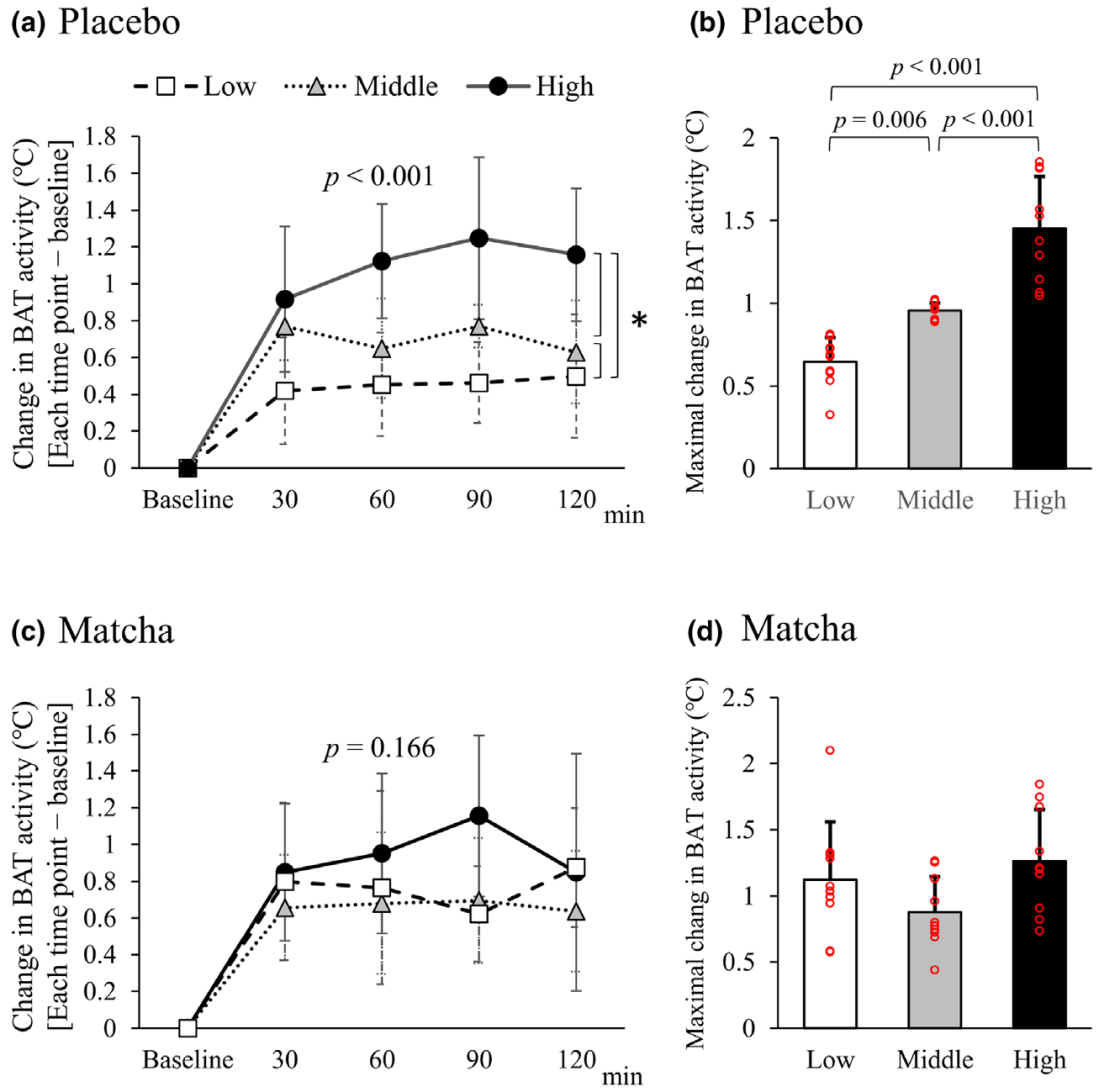
Change in BAT activity in tertile groups according to maximal change in (⊿) BAT activity (⊿ BAT activity). The tertile groups were based on maximal ⊿ BAT activity in a placebo trial. (a) Time course of ⊿ BAT activity in the placebo trial. An asterisk indicate significant difference. (b) Maximal ⊿ BAT activity in the placebo trial. The individual data points are indicated by red circles. (c) Time course of ⊿ BAT activity in the matcha trial. (d) Maximal ⊿ BAT activity in the matcha trial. The individual data points are indicated by red circles.

In the placebo trial, the time course of ⊿ BAT activity and the maximal values were significantly different among the low, middle, and high tertile groups (Figure 4a,b). The matcha trial showed no significant difference in ⊿ BAT activity among the tertile groups (Figure 4c). There was no significant difference in maximal ⊿ BAT activity among the tertile groups after matcha intake (Figure 4d).

**FIGURE 5:**
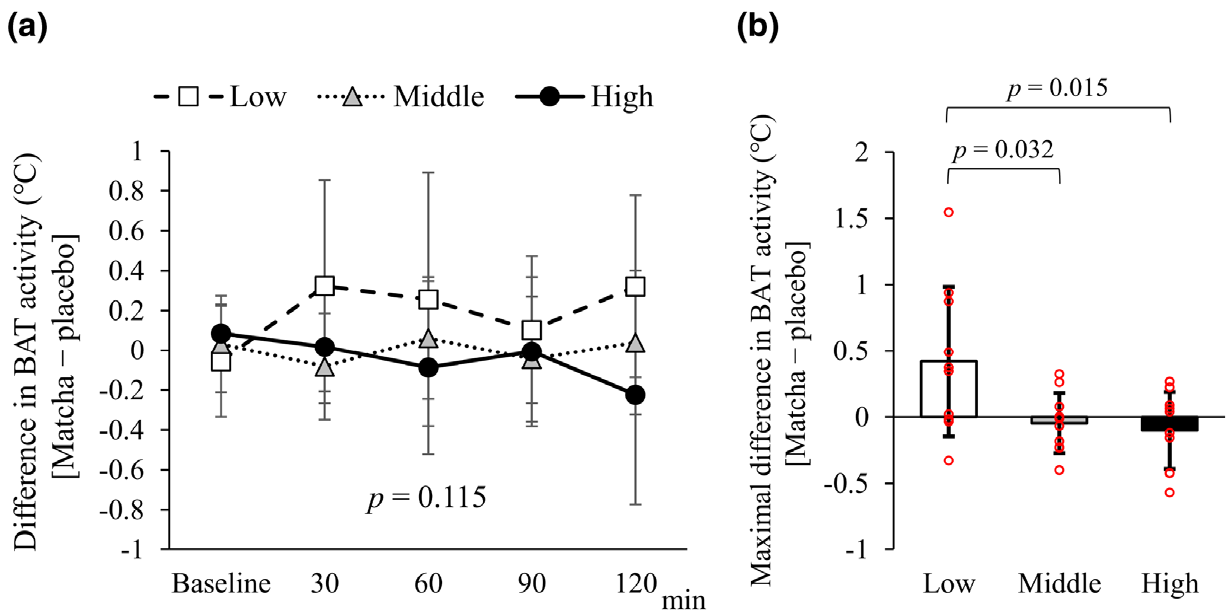
Difference in BAT activity between the matcha and placebo trials among tertile groups according to maximal change in BAT activity. The tertile groups were based on maximal changes in BAT activity in a placebo trial. (a) Time course of difference in BAT activity. (b) Maximal difference in BAT activity. The individual data points are indicated by red circles.

Differences in BAT activity were calculated by subtracting the values from the placebo trial from the matcha trial (Figure 5). Two‐way ANOVA revealed no significant differences in BAT activity among the tertile groups (Figure 5a). The maximal differences in BAT activity were significantly higher in the low activity group than in the middle and high activity groups (Figure 5b).

## 4 DISCUSSION

We performed a randomized crossover study that aimed to determine the effects of single matcha intake on BAT activity in healthy young women. We found no significant differences in BAT activity during cold exposure between the matcha and placebo trials. On the other hand, a stratification analysis by ⊿ BAT activity showed that young women with lower BAT activity had higher levels of BAT activity in the matcha trial than the placebo trial. These results suggest that BAT activation in response to matcha intake does not occur uniformly among young women.

Analysis of the matcha powder used in the present study showed that the amounts of bioactive components, such as catechins, caffeine, and theanine, per unit of weight were consistent with previous studies (Sokary et al., 2023; Ye et al., 2024). A previous study reported an increase in energy expenditure after intake of a beverage containing 615 mg of catechins in individuals with high BAT activity, suggesting that catechins enhance BAT‐derived energy expenditure (Yoneshiro et al., 2017). However, the present study found that matcha had minimal effects on BAT activation. One possible explanation for this discrepancy is the difference in catechin concentration, as the matcha used in the previous study had approximately twofold higher catechin content than the present study (283 mg of total catechins). Further studies are needed to determine whether the intake of tea ingredients activates BAT and thereby increases whole‐body thermogenic indices.

Previous studies reported that single intake of bioactive compounds, including catechins and capsinoids, increased energy expenditure in young men with higher BAT mass but not in those with lower BAT mass (Yoneshiro et al., 2012, 2017). These results suggest that thermogenic capacity in individuals influences the effects of bioactive compounds on BAT activity. Therefore, the present study performed a stratified analysis based on maximal ⊿ BAT activity. In this study, high BAT activity in the placebo trial was not associated with favorable metabolic parameters. The metabolic homogeneity of the healthy participants may explain the absence of significant differences in metabolic parameters among the tertile groups. Comparison among the tertile groups revealed that high levels of BAT activity in the placebo trial were not replicated in the matcha trial. Moreover, the stratified analysis revealed higher absolute and maximal ⊿ BAT activity in the first tertile group, which exhibited lower BAT activity in the placebo trial. Thus, the amount of matcha consumed may be adequate to restore BAT activity but inadequate to enhance BAT thermogenesis.

The present study has several limitations. The participants of the study were exclusively healthy young females, and the sample size was relatively small. The external validity of the present study should be evaluated in other sex and age groups. Furthermore, BAT activity was not assessed using 18 FDG‐PET/CT (Kajimura & Saito, 2014; Sidossis & Kajimura, 2015). Further studies are necessary to evaluate the effects of daily matcha intake on BAT thermogenesis using these modalities.

The present randomized crossover study suggests that a single intake of matcha powder has beneficial effects on the thermogenesis of BAT in young women with lower BAT activity. However, the activation of BAT thermogenesis by matcha intake was not universally observed in all the participants. This suggests that the effect of 3 g of matcha on BAT thermogenesis varies depending on individual characteristics.

## AUTHOR CONTRIBUTIONS

**Hirokazu Taniguchi:** Conceptualization; data curation; formal analysis; funding acquisition; investigation; methodology; project administration; software; supervision. **Chisaki Hachikawa:** Data curation; formal analysis; investigation. **Sana Iwase:** Data curation; formal analysis. **Shinsuke Nirengi:** Conceptualization; methodology; software; supervision. **Tomomi Nagahata:** Data curation; formal analysis; investigation; methodology; project administration; supervision.

## FUNDING INFORMATION

This study was conducted as grant research supported by Matcha and Health Research and supported by JSPS KAKENHI Grant Number 23K10849.

## CONFLICT OF INTEREST STATEMENT

The authors declare that they have no conflict of interest. Matcha and Health Research was not involved in the study's design, data analysis, or preparation of the manuscript.

## ETHICS STATEMENT

All participants provided written informed consent, which was approved by the Ethical Committee of Kyoto Prefectural University (approval number 330). The study was conducted in accordance with the Declaration of Helsinki.

## CODE AVAILABILITY STATEMENT

This study did not use any custom code or software that is not publicly available.

## PATIENT CONSENT STATEMENT

All participants signed an informed consent statement prior to participation in the study.

## PERMISSION TO REPRODUCE MATERIAL FROM OTHER SOURCES

Not applicable.

## Data Availability

The datasets used and/or analyzed during the current study are available from the corresponding author on reasonable request.
